# Moving beyond pain scores: Multidimensional pain assessment is essential for adequate pain management after surgery

**DOI:** 10.1371/journal.pone.0177345

**Published:** 2017-05-10

**Authors:** Regina L. M. van Boekel, Kris C. P. Vissers, Rob van der Sande, Ewald Bronkhorst, Jos G. C. Lerou, Monique A. H. Steegers

**Affiliations:** 1 Department of Anesthesiology Pain and Palliative Medicine, Radboud University Medical Center, Nijmegen, The Netherlands; 2 Faculty of Health, HAN University of Applied Sciences, Nijmegen, The Netherlands; 3 Department of Medical Statistics, Radboud University Medical Center, Nijmegen, The Netherlands; Université catholique de Louvain, BELGIUM

## Abstract

**Background:**

Clinical experience teaches us that patients are willing to accept postoperative pain, despite high pain intensity scores. Nevertheless, relationships between pain scores and other methods of pain assessment, e.g. acceptability of pain or its interference with physical functioning, are not fully established. Our aims were to examine these relationships.

**Methods:**

A cross-sectional study was conducted on patients who underwent major surgery between January 2008 and August 2013. Using logistic regression, we quantified the relationships between movement-evoked pain scores on the numerical rating scale (NRS-MEP) and three dichotomous dependent variables: patient’s opinion on acceptability of pain (PO: acceptable or unacceptable pain); nurses’ observation of patient’s performance of necessary activities to expedite recovery (NO: good or bad performance); a compound measure judging the presence of the clinically desirable situation of acceptable pain associated with good patients’ performance (PONO: present or not). Using Receiver Operating Characteristics (ROC) analysis, NRS cut-off points were determined such that they best discriminate between patients having one versus the other outcome for PO, NO and PONO.

**Results:**

15,394 assessments were obtained in 9,082 patients in the first three postoperative days. Nine percent of the patients had unacceptable pain while having an NRS-MEP of 0–4. An estimated 47% (95%CI = 45%-49%) of patients with an NRS-MEP of 7 described their pain as acceptable on day one. Moreover, 33% (31%-35%) performed all required physical activities, and 22% (21%-24%) combined acceptable pain with appropriate movement. NRS cut-off points for PO, NO and PONO were five, four and four, respectively, but had insufficient discriminatory power.

**Conclusions:**

Our results suggest pain management should be guided by the many dimensions of the patient’s pain experience, not solely by NRS cut-off points. Future research should evaluate the impact of such multidimensional pain assessment on patients’ functional outcome.

## Introduction

Many patients experience acute postoperative pain after major as well as minor surgery [1, 2]. Clinical experience teaches us that really adequate treatment of postoperative pain is not easy to achieve. To balance treatment options, treatment starts with assessing the pain. As pain is a complex and subjective experience, also in the postoperative period, various methods exist to evaluate key aspects of acute pain after surgery.

Most of these assessment methods rely on the perception of pain and pain- related phenomena by either the patient or a professional caregiver [3, 4]. Self-assessment of pain by the patient may use a pain intensity scale and yes/no answers to questions such as “Is the pain acceptable?” [5, 6]. Self-reporting values the subjective nature of pain. Evaluation of pain by a professional may include objective assessment of the functional impact of pain. The professional therefore judges if the pain prevents the patient from moving appropriately or from performing the necessary activities to expedite recovery [7]. One clinically important goal could be a level of pain that is not only acceptable for the patient, but also allows the patient to move appropriately as judged by a professional.

The numerical rating scale (NRS), a validated instrument to assess pain intensity by self-reporting, is widely used for assessing pain on a scale from zero (no pain at all) to 10 (worst possible pain). Certain NRS scores have even been used as cut-off points to guide initiation or cessation of treatment in an individual or even as indicator of the quality of pain management in a population [8–10].

Relationships between NRS and other methods of pain assessment, e.g. acceptability of the pain or its interference with physical functioning, are not fully established. In the clinical setting, some patients report a high movement-evoked pain score, yet claim that their pain is acceptable to them [11]. Patients may even refuse to take pain medication when an NRS cut-off point demanding treatment according to a pain protocol is reached or crossed [12]. A further complicating factor is that some patients and pain professionals interpret pain scores differently [3]. As a result of these discrepancies or unclear relationships between different pain assessments, difficulties in treatment decisions may arise.

Our aims therefore were *first*, to quantify relationships between NRS and other methods of pain assessment and *second*, to examine the ability of an NRS cut-off point to predict either patients’ willingness to accept pain or functional capacity. Potential benefit of the study is that its results may aid to develop and corroborate clinical guidelines to tailor postoperative pain management in a way that will meet the unique needs of each patient.

## Materials and methods

### Approval

The Institutional Review Board of the Radboud University Medical Center (Nijmegen, The Netherlands) approved the study (2013/428). No informed consent was obtained from the participants because data were anonymized.

### Study design and patients

This cross-sectional study was conducted on patients older than 18 years who had been admitted in a large regional academic medical center in the period from 1 January 2008 to 1 August 2013. The study used the prospectively collected pain assessments of postoperative patients who had been treated by the acute pain service (APS).

We quantified the relationships between movement-evoked NRS and acceptability of pain, functional impact of pain, and a measure combining the two. The latter measure serves to judge whether or not a clinically desirable situation occurs where acceptable pain coexists with good physical functioning. A potential influence of gender, age or body mass index (BMI) was investigated.

### Data handling

#### Assessments

The APS nurses use a standardized multidimensional assessment to evaluate postoperative pain. This assessment includes: (1) the NRS for movement-evoked pain (NRS-MEP) [7], (2) the patient’s opinion (PO) whether the pain is acceptable because the patient’s appreciation of the pain is clinically important for making the patient comfortable [13], and (3) the nurses’ observation (NO) on the patient’s ability to make appropriate movements. NRS-MEP and NO are important because adequate treatment of pain experienced during pain-provoking maneuvers may reduce complications after surgery [14, 15].

The NRS-MEP is an 11-point numerical rating scale with end points representing the extremes of the pain experience: 0 = “no pain at all” and 10 = “worst possible pain”. All nurses and patients received education on how to use the NRS-MEP appropriately [15].

The PO is determined by asking the patient whether the pain is acceptable or not, making it a binary yes-or-no variable [11].

The NO scoring mirrors the Functional Activity Score (FAS) described by Scott and McDonald [16] and adopted by the Australian and New Zealand College of Anaesthetists. The FAS, recommended in several textbooks [17, 18], was recently integrated in the updated Australian and New Zealand guideline on acute pain management [19]. The FAS (designed to be applied at the bedside) is a simple three-level ranked categorical score to assess whether the patient can undertake appropriate activity at his or her current level of pain control.

The APS nurses rely on an operation-specific protocol offering clearly defined criteria to judge patient’s ability to perform physical activities on the first three days after surgery—like coughing, deep breathing, early movement and walking [20]. Some examples of operation-specific protocols are the ability to sit on a chair for thirty minutes on the first morning after a patient has had a laparotomy and the ability to walk to the bathroom for a patient on the first day after a total hip replacement. Patient’s performance is qualified as: “good”, “moderate” or “bad”. A “good” means patient is able to make all appropriate movements and is not hindered by pain. “Bad” means patient is totally unable to make appropriate movements because of the pain. “Moderate” is chosen when observing neither “good” nor “bad”. The results for NO are dichotomized into two outcome categories, “good” or “moderate and bad”. Accordingly, NO is also a binary yes-or-no variable.

In addition, combining PO and NO yields a third binary yes-or-no variable, i.e. PONO. This variable is not part of the multidimensional assessment at the bedside, but was created for study purposes only. One result for PONO is when “acceptable pain” accompanies “good movements”, thus reflecting a clinically desirable situation. The ultimate goal of postoperative pain treatment is that a patient qualifies the pain as acceptable and is able to perform appropriate movements. The other result for PONO is chosen for each of the three remaining combinations of PO and NO.

#### Database of acute pain service

The nurse-based, anesthesiologist supervised APS is part of the Department of Anesthesiology, Pain and Palliative Medicine. The organization of this type of APS has been described elsewhere [21, 22]. The APS has a team of five dedicated well-trained nurses who strictly use hospital protocols to assess postoperative pain in patients. The APS is available seven days a week and supports the treatment of postoperative pain with specialized or complex pain management techniques. The APS treats patients from the first day after major surgery, but not on the day of surgery. Typical surgical procedures are listed in the supplementary information (see S1 Table).

After each visit to a patient, the dedicated nurse enters the obtained data (*inter alia* values for NRS-MEP, PO and NO) into a new digital record of the APS database. As each visit yields one record in the database, multiple records per patient are possible per day. Data are entered on a mobile handheld computer wirelessly connected with the hospital system for real time registration.

Standard information about postoperative pain and its management is given preoperatively to patients. The information is recorded in a pain protocol. Anesthesiologists give oral information supported by a leaflet during the preoperative consultations. Patients are also invited to watch a movie online. Prior to the actual pain assessment, APS nurses check patient’s knowledge and, if necessary, still inform the patient using the appropriate information.

#### Creating data sets

**Fig 1:**
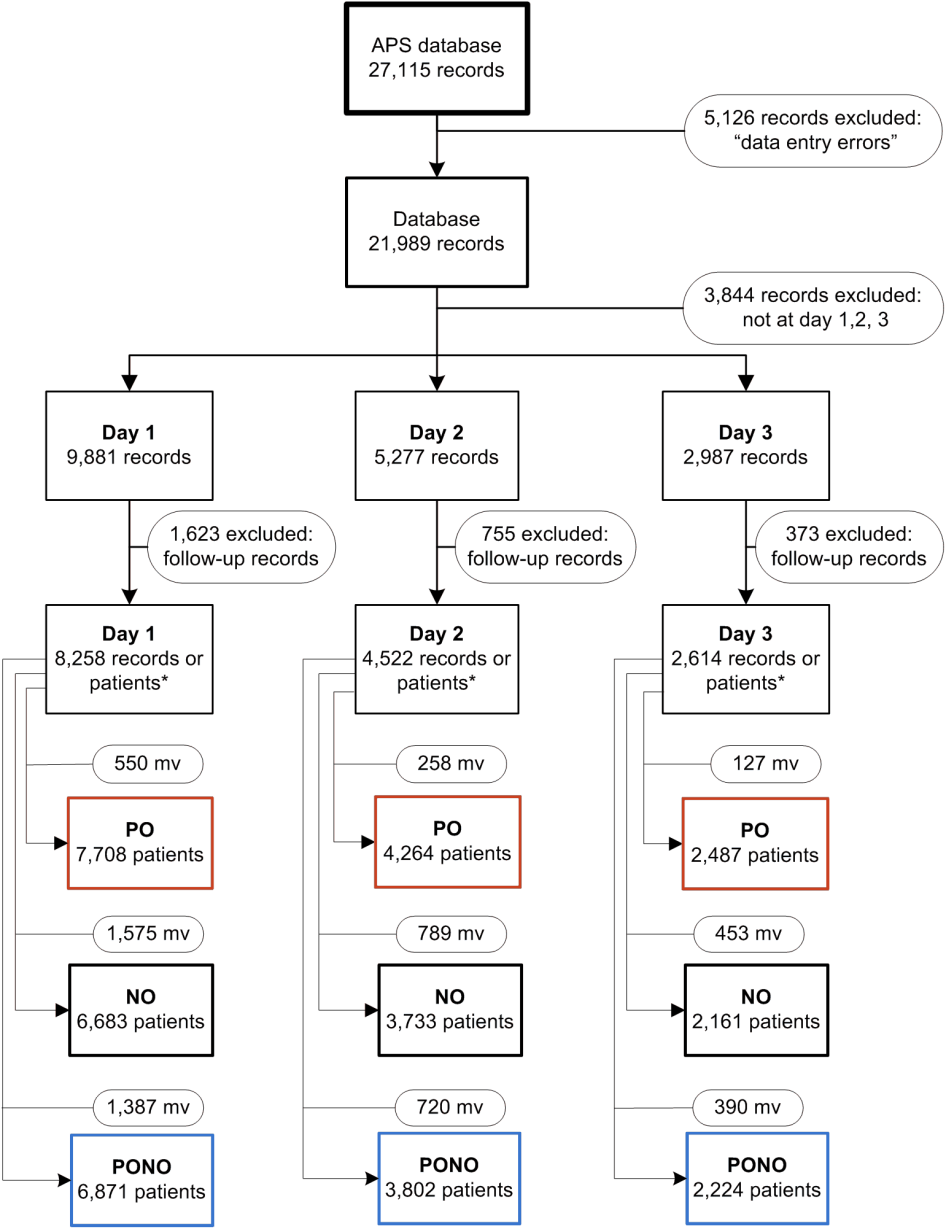
Flow chart: Transforming the database of the Acute Pain Service (APS) into nine data sets. For each of the three postoperative days, three data sets are created: one for patient’s opinion (PO), one for nurses’ observation (NO) and one for the combined variable (PONO). *Before this point multiple records are possible per patient but after this point the number of records equals the number of patients. mv = missing values.

Fig 1 illustrates the various steps to create datasets ready for valid analyses from the APS database. Editing the raw APS database was necessary because of data entry errors. Data entry errors were found in records with pain scores above ten and records where pain scores were not entered or patients were unable to give an NRS score. As multiple records per patient were possible per day for many days in a row, we made a selection *first*, by taking the records of the visits of the first three days after surgery and *second*, by selecting the record of the first visit to a patient per day to stay in the database. As a result, the number of records equaled the number of patients on day 1, day 2, and day 3 after surgery.

As the APS database consists of the real time online registration during the work of the APS nursing staff, some missing values were also inevitable. These missing values were counted per day for the PO-, NO- and PONO-variables (Fig 1). To avoid the bias that would be induced by restricting the analyses to patients without missing observations, we did not exclude patients because of incompleteness of the pain assessments.

### Statistical analysis

To explore the relationships between PO, NO, PONO and NRS-MEP, the relative frequencies of the two possible outcomes for PO, NO, and PONO during the first three postoperative days were pooled and were plotted against the NRS for MEP.

To quantify these relationships, a logistic regression model was estimated using the 11-point NRS for MEP as primary independent explanatory variable for each of the three dependent variables PO, NO, and PONO. Thus PO, NO, and PONO served as gold standards. As gender, age and BMI may influence the results, these patient characteristics were introduced as extra dichotomous explanatory variables (covariates) into the logistic model [9, 23–25]. Details on the model variables are given in Table 1. A model was calculated for each of the three postoperative days.

**Table 1:** Name, abbreviation, values and coding of variables used in the logistic regression models to estimate the relationships between four explanatory variables and each of three response variables.

	Variable name	Abbreviation	Values	Coding
**Explanatory variables**	Numerical Rating Scale	NRS	0−10	0 = no pain
				10 = worst pain imaginable
	Age	A	0 or 1	0 = younger than 65 years
				1 = 65 years or older
	Gender	G	0 or 1	0 = female
				1 = male
	Body mass index	BMI	0 or 1	0 = BMI < 30 kg m^-2^
				1 = BMI ≥ 30 kg m^-2^
**Response variables**	Patient’s opinion	PO	0 or 1	0 = pain is not acceptable
(One per model)				1 = pain is acceptable
	Nurses’ observation	NO	0 or 1	0 = no appropriate movement
				1 = appropriate movement
	Combined PO+NO	PONO	0 or 1	1 = PO = 1 and NO = 1
				0 = otherwise

Receiver Operating Characteristics (ROC) curves were made to estimate the ability of the computed models to correctly discriminate between those who found their pain acceptable or not, made appropriate movements or not, and those who combined acceptable pain with appropriate movements or not. First the sensitivity and specificity of NRS-MEP were calculated for each of the 11 points of the NRS-MEP score. Then the sensitivities (true positive fractions of subjects) were plotted versus 1-specificities (false positive fractions of subjects) to obtain the ROC curves. The area under the curve (AUC) quantifies how well the NRS-MEP predicts PO, NO or PONO: the larger the area, the better. If AUC = 1.0, sensitivity and specificity equal both 100%. If AUC = 0.5, use of NRS-MEP is no better than flipping a coin.

The statistically optimal cut-off point was determined where the sum of the sensitivity and the specificity minus one (Youden’s J-statistic) was maximal. Thus sensitivity and specificity were regarded as being equally important. This is the best cut-off point for the prediction of a positive response under the condition of equal “costs” of misclassifications.

The Statistical Package for the Social Sciences (IBM SPSS version 22.0; IBM Corporation, New York, NY, USA), Statistical Analysis System (SAS version 9.2; SAS Institute Inc., Cary, NC, USA), and R (R version 3.1.2 (2014-10-31); The R Foundation for Statistical Computing, Vienna, Austria) were used. Threshold of statistical significance was 0.05.

## Results

### Patient characteristics

15,394 assessments were obtained in 9,082 unique individual patients. For each of these patients data were obtained on: one of the three postoperative days, or any combination of two days, or all three days. Consequently, we had data from these 9,082 individual patients for 8,258, 4,522 and 2,614 of them on day 1, day 2 or day 3, respectively (Fig 1). The number of patients diminished across the three days as a part of the patients left the hospital after one or two days. A detailed account of the numbers of patients and assessments is given in S2 Table (see S2 Table, which is a comprehensive table listing the number of patients, the number of unique, individual patients and the number of assessments of patients categorized per day or per combination of days).

Table 2 shows patients’ characteristics categorized per day. They were similar when further categorized per data set.

**Table 2:** Numbers and characteristics of patients.

Day after surgery	n	Male (%)	Age (years) (mean (SD))	BMI (kg m^-2^) (mean (SD))*
1	8,258	44.0	53.5 (16.3)	26.2 (4.9)
2	4,522	51.5	56.5 (15.4)	25.8 (4.7)
3	2,614	55.5	56.8 (15.3)	25.7 (4.6)

* Because of missing values for length and/or weight the means (SD) for BMI are based on 8,042, 4,406, and 2,546 patients for day 1, day 2 and day 3, respectively.

### Relationships between components of pain assessment

#### Observations

**Fig 2:**
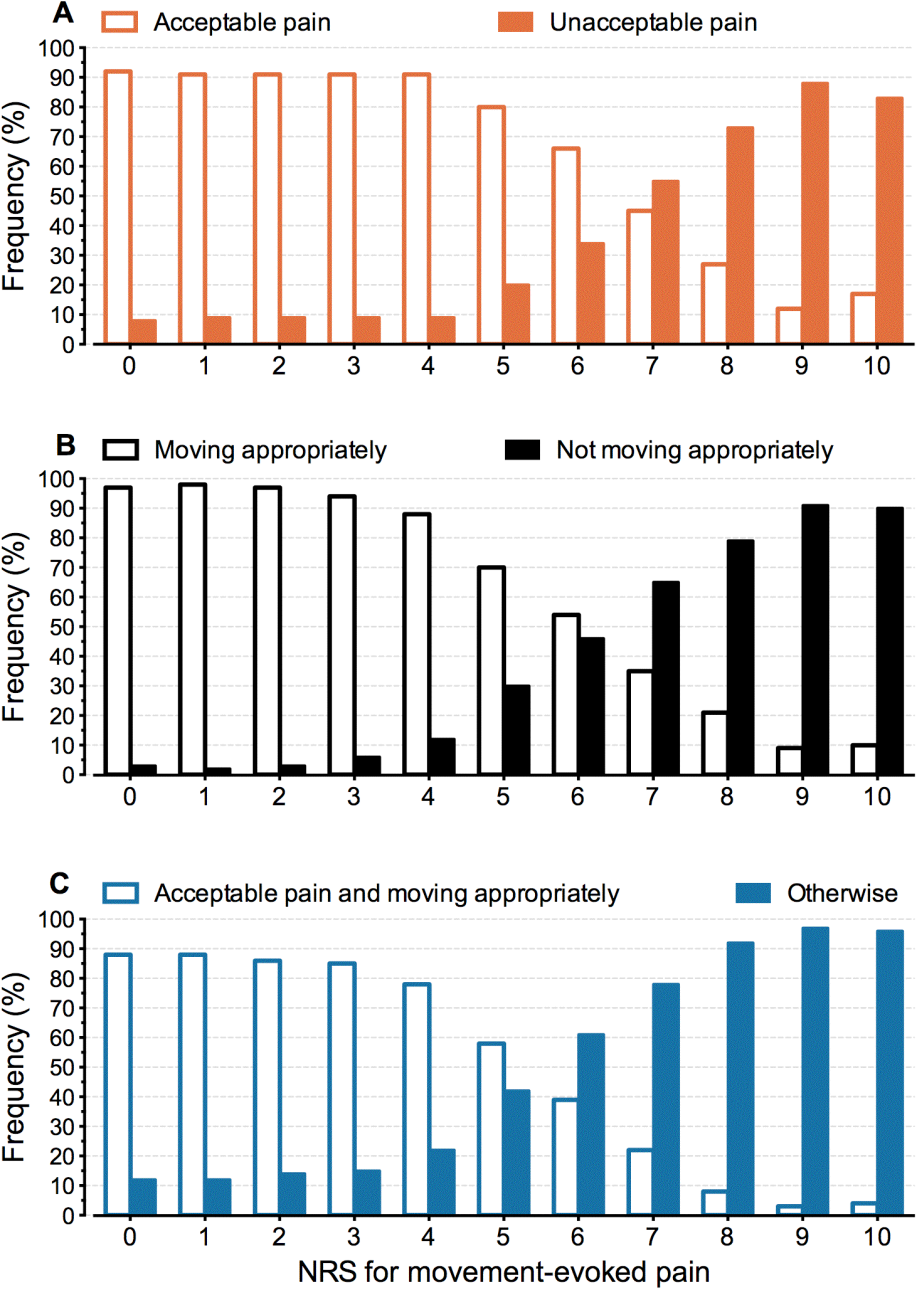
**Relative frequencies for observations of patients’ opinion (A), nurses’ observation (B), and the measure combining patient’s opinion and nurses’ observation (C) against NRS-MEP scores.** The observations in all patients gathered during the first three postoperative days were pooled.

Fig 2 depicts the nature of the relationships between components of pain assessment. Pooled observed relative frequencies for PO, NO and PONO are plotted against NRS-MEP scores. The sigmoid shape of the relationships suggests using a logistic model for further analysis.

Fig 2A shows the observed relationship between the NRS-MEP scores and the acceptability of the pain. Patients associated low NRS-MEP scores 0–4 with unacceptable pain in approximately 9% of the observations. On average, in 23% of the observations patients with an NRS-MEP of 8–10 considered their pain acceptable.

Fig 2B shows that, on average, in 17% of the observations patients with an NRS-MEP of 8–10 showed appropriate movements.

Fig 2C shows the observed relationship between the NRS-MEP scores and the presence of a clinically desirable situation where acceptable pain coexists with pain-free physical functioning. This situation is present in 22% of the observations with an NRS-MEP = 7, and, on average, in 7% of the observations with an NRS-MEP of 8–10.

### Model-based relationships

Binary logistic regression analysis revealed strong mathematical relationships between components of pain assessments, but age, gender and BMI were of no influence. All fitted models were adequate (likelihood ratio statistic: all P- values < 0.001).

NRS-MEP was related to PO, NO or PONO on each of the three postoperative days (all P-values < 0.001). The procedure to assess the influence of age, gender and BMI on the prediction models yielded 27 P-values (three days times three covariates times three response variables). Of these 27 P-values, only one was below the threshold of statistical significance (P = 0.0423 for gender on the prediction of NO on day 1). As even in the absence of any relation between these covariates and the three outcomes, by pure chance one out of 20 P-values can be expected to be <0.05, these P-values were interpreted as indication that extension of the models with these covariates was not indicated. Therefore, we only present analyses using NRS-MEP as sole covariate. Details on the estimated models, including estimated regression coefficients, odds ratios and ROC curves, are given in S1 Fig (see S1 Fig).

**Fig 3:**
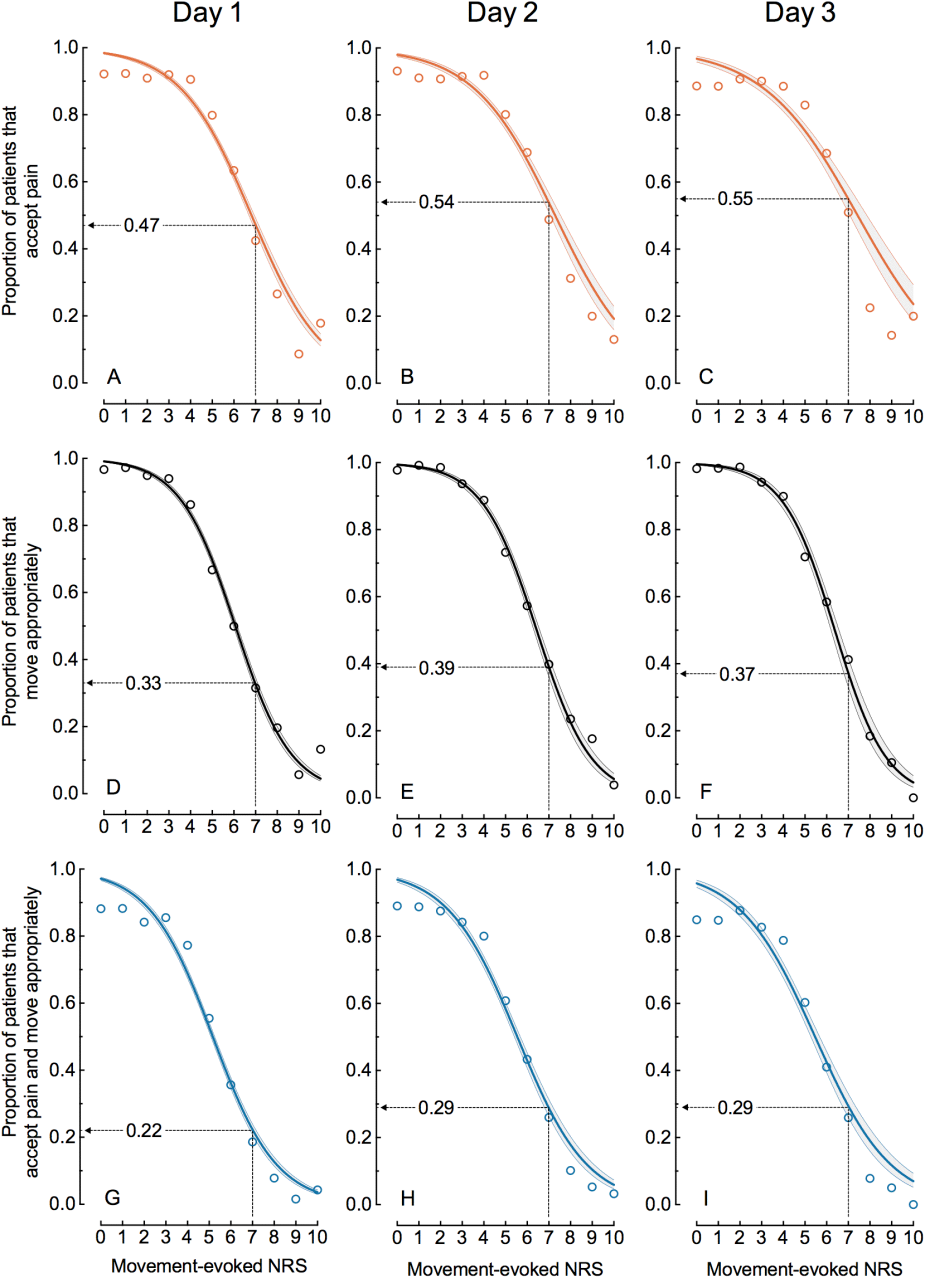
**Estimated proportion (solid curve with its 95% confidence band) of patients that accept the pain (A-C), move appropriately (D-F) or accept the pain and move appropriately (G-I) as a function of NRS-MEP for day 1, 2 and 3 after surgery.** The open circles represent the observed proportions of patients at each of the eleven points of the NRS. For each of the nine data sets, one estimated proportion is computed and shown at NRS-MEP = 7.

Fig 3 shows the estimated logistic curves with their 95% confidence bands for the nine data sets created as shown in Fig 1. Wider 95% confidence bands reflect smaller numbers of patients. Each of the curves shows the estimated proportion of patients that possess the outcome measure of interest as a function of NRS-MEP [26]. The estimated curves for NO strongly match the data for nurses’ observations indicated by open circles. For PO and PONO the curves closely follow observed proportions for 3≤NRS≤8 but mostly overpredict the observations for NRS≤2 and NRS≥9.

Fig 3 shows that, despite an NRS-MEP = 7, roughly half of the patients accept the pain (Fig 3A–3C) and at least one third of the patients move appropriately (Fig 3D–3F). Fig 3 suggests that these proportions increase with time. In spite of an NRS-MEP = 7, at least one patient in five finds the pain acceptable and moves appropriately (Fig 3G–3I): estimated proportions are 0.22 (95% CI = 0.21–0.24), 0.29 (95% CI = 0.26–0.31) and 0.29 (95% CI = 0.26–0.33) for day 1, 2 and 3, respectively.

**Fig 4:**
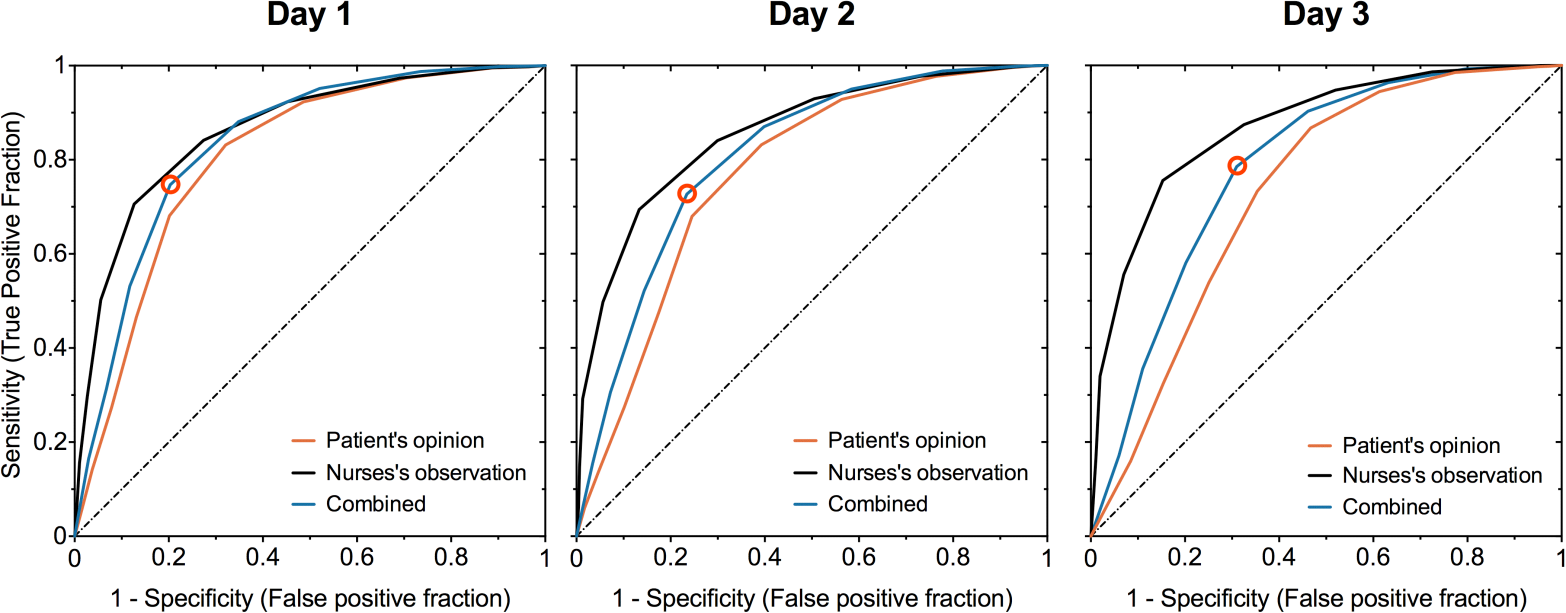
ROC curves for the dependent variables PO, NO and PONO for the three first postoperative days. The dashed line is the line of identity where the AUC = 0.5. Open circles are the points where Youden’s J-statistic is maximal for PONO. These points are, by definition, the ‘optimal’ cut-off points.

Table 3 presents the statistically optimal cut-off points with their associated sensitivities and specificities. The number of patients decreases across the days. The cut-off points, however, remain stable: five, four, and four for PO, NO and PONO, respectively. Fig 4 shows graphs of the ROC curves. The closer a ROC curve is to the upper left corner, the better NRS-MEP discriminates between those patients who experience the outcome of interest, e.g. the pain is acceptable, versus those who do not.

**Table 3:** Cut-off points obtained from the logistic regression model using the 11-point Numerical Rating Scale for movement-evoked pain as explanatory variable for each of the three dependent variables PO, NO, and PONO. Shown are the optimal cut-off points with their associated sensitivities and specificities, as well as the areas under the ROC curves (AUC).

Day after surgery	Dependent variable	N	Cut-off point	Sensitivity(%)	Specificity (%)	AUC	(95% CI)
1	PO	7,708	5	83	68	0.81	(0.79–0.82)
	NO	6,683	4	71	87	0.86	(0.85–0.87)
	PONO	6,871	4	75	80	0.84	(0.83–0.84)
2	PO	4,264	5	83	61	0.77	(0.75–0.79)
	NO	3,733	4	69	87	0.86	(0.84–0.87)
	PONO	3,802	4	73	77	0.81	(0.79–0.82)
3	PO	2,487	5	87	53	0.73	(0.71–0.76)
	NO	2,161	4	76	85	0.87	(0.85–0.89)
	PONO	2,224	4	79	69	0.79	(0.76–0.81)

PO, patient’s opinion on whether the pain is acceptable; NO, nurses’ observation on the patient’s ability to make appropriate movements; PONO, combined measure of PO and NO: is “acceptable pain” associated with “good appropriate movements" or not. Details on PO, NO, and PONO are given in Table 1.

The AUC for PO decreases across the days from 0.81 to 0.73. The latter figures indicate that NRS-MEP is not a perfect predictor for patients’ willingness to accept their pain. The areas under the curve for NO are larger than those for PO and PONO on each of the three days. The AUC for NO implies that the NRS-MEP is fairly accurate in predicting the NO for all three days [27].

Four is the statistically optimal cut-off point for NRS-MEP based on the combination of patients’ opinion and the nurses’ observation. Nevertheless, 17%, 15% and 17% of those patients, who scored an NRS-MEP≤4, found their pain unacceptable or did not show good physical functioning or both, on day 1, 2, and 3, respectively. Fig 3G shows that the steepest part of the sigmoid curve starts at the cut-off point (odds ratio = 0.51 with its 95%CI = 0.49–0.52 for day 1; other odds ratios are given in S1 Fig (see S1 Fig).

## Discussion

To our knowledge, this is the first study in a broad surgical population to quantify the relationships between movement-evoked NRS and acceptability of pain, functional impact of pain, and a measure combining the two as a clinically desirable situation. Since the outcome of pain assessments has clinical consequences for all surgical patients, we consider our findings important to all health professionals involved in peri-operative care.

This study shows that the unidimensional NRS does not entirely reflect the multidimensional aspects of postoperative pain. Low pain scores do not guarantee that patients find their pain acceptable. Nor do high pain scores invariably mean that patients are not satisfied by their pain levels. Approximately one out of ten patients had unacceptable pain but reported a low NRS-MEP of 0–4. Despite a high pain score of NRS-MEP = 7, at least one in five patients were willing to accept their pain and, at the same time, performed the required physical activities (Fig 3G–3I).

According to the Youden’s index, we found an ‘optimal’ NRS cut-off point for PONO of four. However, this threshold value is a rather poor predictor at the patient’s level. Approximately 16% of those patients who score an NRS-MEP equal to or lower than four, found their pain unacceptable or did not show good physical functioning or both. Taken together, the body of our findings points out that caregivers should prefer multidimensional assessment of pain, moving beyond the sole use of cut-off points on the NRS to make clinical decisions.

Generally, low pain scores will not encourage health professionals to adjust pain treatment [28]. When health professionals do not ask patients whether pain is acceptable to them, pain may be undertreated. On the other hand, our study confirms the willingness of many patients to accept high-intensity pain. Maroney and co-workers observed that 31 percent of 1,249 patients, who reported severe pain on a four-item scale, found their pain acceptable [11]. In our larger study 23% of patients, on average, proved to tolerate their pain despite an NRS-MEP of 8–10 (Fig 2A). At NRS-MEP = 7, the estimated proportion of patients tolerating their pain was even 55% (95%CI = 51%-59%) on the third postoperative day (Fig 3C). These discrepancies may be explained by patients’ satisfaction with postoperative pain treatment, which may be more associated with impressions of improvement and appropriateness of care than with the actual pain experience [29, 30]. Additionally, patients and caregivers interpret pain intensity scores differently [3]. A recent study showed that some patients are not able to use the NRS reliably [31]. Patients may choose not to take more analgesics because they interpret their pain as “bearable” [12, 32]. Professionals need to be aware of this complex array of factors determining patients’ experience of the pain. Therefore, the patient perspective should be assessed and valued in the care process [29].

To fully estimate patients’ experience of pain an NRS score is not sufficient and other dimensions of pain should be assessed to balance treatment options [33, 34]. The internationally recognized definition by the International Association for the Study of Pain is: “Pain is an unpleasant sensory and emotional experience associated with actual or potential tissue damage, or described in terms of such damage” [35]. McCaffrey and Beebe offer another definition:"Pain is whatever the experiencing person says it is, existing whenever the experiencing person says it does" [36]. Both of these definitions highlight that a painful experience is more than just tissue damage triggering a response from the nervous system. The management of pain thus involves more than simply treating the tissue injury [37–39]. NRS-scores should be interpreted individually, after communicating with patients about their pain and observing them [12]. Observing the capacity to mobilize, breathe deeply or cough may inform the professional on the functional capacity of the patient in relation with the pain score [7]. Restrictions of these activities may be a consequence of inadequate analgesia, which may not be discovered solely with patient-reported outcomes [4].

As nurses have more patient contacts than other health professionals, regular pain assessment and reassessments usually fall to the nursing domain [40]. Pain assessment is a complex communication process between the patient and health professional with diverse interpersonal and intrapersonal dimensions interacting and affecting each other [13, 41]. In this way a balanced decision on pain treatment can be described as the result of a social transaction between the patient and the health professional [13, 42]. The combination of the patients’ opinion and the nurses’ observation, as the balancing variable, may therefore be a first step in the direction of the future.

A specified value on the NRS has been frequently used as a single ‘cut-off point’ to divide patients into two categories: those who are in need of pain treatment and those who are not [8]. However, cut-off points are far from perfect discriminators between the two categories (Table 3). Also, there is no convincing evidence for the choice of a certain cut-off point, and consequently no consensus [8, 12, 15]. Threshold values of six [9], seven [15], or eight have been used to define the lower limit for severe pain. The Dutch Health Care Inspectorate classifies NRS ≥ 8 as severe pain and considers the percentage of patients with an NRS 8–10 to be a quality indicator of postoperative pain management [10]. Furthermore, there is no evidence that the use of cut-off points improves pain control [13].

The ‚optimal’ cut-off point for NRS-MEP we defined here holds under the condition that costs of misclassifications are equal, thus weighing under- and over-treatment equally. However, our choice for this equality cannot be corroborated because it is unknown what is more harmful. In this study no outcome data were included and therefore we cannot discuss our results from this perspective. Nevertheless, we may point out two directions for future research. On one hand, questions should be answered whether treating unacceptable pain and better education of patients and professionals may prevent pain-related complications [14]. On the other hand, a hypothesis to be tested is: „Treating pain during routine hospital ward care, only because a pain score is higher than a predefined cut-off value, is potentially hazardous”.

In our study, we did not exclude patients because of incompleteness of the pain assessments. By doing so, we avoid the bias that would be induced by restricting the analyses to only patients without missing observations, the so-called complete case analysis. A complete case analysis is unbiased if data are missing completely randomly, meaning that the chance of data being missing is unrelated to any of the variables involved in the analysis. If data are not missing completely randomly, analyzing only the complete cases will probably lead to biased estimates [43]. Even when complete case analysis would be unbiased, discarding all the information from the incomplete cases is inefficient.

This study has limitations. First, there are no "gold standard" objective measures of the pain-related functional capacity in postsurgical patients [44]. Nevertheless, various measures have been developed to quantify treatment related changes in the physical abilities of individuals with acute pain [4, 16]. The FAS is such a nonvalidated—because of lacking standards—measure. Not only has the FAS been adopted by the Australian and New Zealand College of Anaesthetists and Faculty of Pain Medicine [19], but also it has been advocated for clinical use [16–18]. The FAS proved to be very useful and generally applicable in daily practice. Second, we could not include all confounding factors. Gender, age and BMI were introduced as covariates in the logistic model because they are risk factors for the development of acute postoperative pain [9, 23–25]. Gender, age and BMI showed no influence, but we do not know if other factors might. Other factors may be: type of operation, anxiety or catastrophizing [9, 45], preoperative information, expectations about pain levels, psychological profile and motivation. The impact of these factors with the relationships between NRS-MEP, PO, NO and PONO could be a topic of future prospective studies. For example, pain anticipation can be assessed by asking the patient preoperatively to mark a point on the NRS that describes the anticipated pain after surgery [46]. Third, our findings do not apply to all hospitalized patients because we only studied patients after major surgery. One next step is to validate our results for other patient categories, such as patients after minor surgery and patients with cancer pain.

### Conclusions

The nature and strength of the relationships we found lead to clinically important findings and implications. Almost one in ten patients has unacceptable pain even if they report a low pain score. One in five patients with a high pain score accepts the postoperative pain and still moves appropriately. We encourage health professionals to use a multi-source pain evaluation by assessing NRS, the acceptability of the pain and physical functioning in order to balance pain treatment options and possible complications. The sole use of NRS cut-off points is not adequate. Adequate pain assessment appears to become a form of social transaction between patient and caregiver. Future research should focus on the improvement in pain-related outcomes in relation to multidimensional pain assessment and treatment decisions.

## Supporting information

S1 Table Surgical procedures categorized in ten groups. (PDF)

S2 Table Number of patients, number of unique, individual patients and the number of assessments of patients categorized per day or per combination of days. (PDF)

S1 Fig Results from logistic regression. (PDF)
