# Supplementary material for: Moving beyond pain scores: Multidimensional pain assessment is essential for adequate pain management after surgery
Source: PLoS One. 2017 May 10;12(5):e0177345. doi: 10.1371/journal.pone.0177345 (PMC5425226; doi:10.1371/journal.pone.0177345)
Supplement: S1 Table — (PDF) [file pone.0177345.s001.pdf]

S1 Table. Surgical procedures, categorized in ten groups

Group	N	Procedure
Laparotomy	3,610	Hyperthermic Intraperitoneal Chemotherapy (HIPEC), colectomy, hepatectomy, Whipple procedure (pancreaticoduodenectomy), pelvic or retroperitoneal lymph node dissection, Oscar Ramirez procedure (abdominoplasty), adhesiolysis.
Upper laparotomy	1,857	Gastrectomy, pancreatectomy, splenectomy, esophageal resection, cholecystectomy.
Lower laparotomy	2,792	Abdominal hysterectomy, appendectomy, cystectomy, prostatectomy, sectio caesarea, debulking, rectum or sigmoid resection.
Laparoscopy	581	Laparoscopic cholecystectomy, nephrectomy, colectomy, adrenalectomy, prostatectomy, hysterectomy.
Sternotomy	62	Coronary bypass, thymectomy, metastasectomy, pericard resection.
Thoracotomy	943	Lobectomy, pneumectomy, tracheal resection.
Thoracoscopy	402	Video assisted surgery or thorascopic surgery, such as pleurectomy, bullectomy, Nussbar surgery, video-assisted thorascopic surgery.
Flank	311	Nephrectomy, adrenalectomy.
Orthopaedic surgery	2,966	Total knee arthroplasty, total hip arthroplasty, acetabulum arthroplasty, arthrodesis of limbs, arthrotomy, osteotomy, amputation of limbs, pelvectomy, total shoulder arthroplasty, hand surgery, foot surgery.
Remainder of procedures	1,870	Hernia inguinalis, mastectomy, lumpectomy, laminectomy, spondylodesis, head or face surgery.
