# Supplementary material for: Moving beyond pain scores: Multidimensional pain assessment is essential for adequate pain management after surgery
Source: PLoS One. 2017 May 10;12(5):e0177345. doi: 10.1371/journal.pone.0177345 (PMC5425226; doi:10.1371/journal.pone.0177345)
Supplement: S2 Table — (PDF) [file pone.0177345.s002.pdf]

**S2 Table.** Number of patients, number of unique, individual patients and the number of assessments of patients categorized per day or per combination of days.

| Pain assessment on | Day 1 | Day 2 | Day 3 | Unique patients | Assessments |
|---|---|---|---|---|---|
| Only day 1 | 4,327 | 0 | 0 | 4,327 | 4,327 |
| Only day 2 | 0 | 393 | 0 | 393 | 393 |
| Only day 3 | 0 | 0 | 88 | 88 | 88 |
| Day 1 & 2 | 1,748 | 1,748 | 0 | 1,748 | 3,496 |
| Day 1 & 3 | 145 | 0 | 145 | 145 | 290 |
| Day 2 & 3 | 0 | 343 | 343 | 343 | 686 |
| Day 1 & 2 & 3 | 2,038 | 2,038 | 2,038 | 2,038 | 6,114 |
| **Total** | **8,258** | **4,522** | **2,614** | **9,082** | **15,394** |
