# Supplementary material for: Moving beyond pain scores: Multidimensional pain assessment is essential for adequate pain management after surgery
Source: PLoS One. 2017 May 10;12(5):e0177345. doi: 10.1371/journal.pone.0177345 (PMC5425226; doi:10.1371/journal.pone.0177345)
Supplement: S1 Fig — (PDF) [file pone.0177345.s003.pdf]

S1 Fig. Results from logistic regression.

Using the sample data, an iterative process (maximum likelihood) produces an estimated logistic regression equation of the form

$$logit(p) = log_e \frac{p}{1-p} = a + b_1 NRS + b_2 A + b_3 G + b_4 BMI \qquad (1)$$

where:

- *NRS, A, G* and *BMI* are the explanatory variables (Table 1 in the Methods section);
- *p* is the estimated value of the true probability that a patient with a particular set of values for the explanatory variables has the outcome of interest, for example the patient moves appropriately;
- *a* is the estimated constant term;
- b_1, b_2, b_3, and b_4 are the estimated logistic regression coefficients.

We can manipulate equation (1) to estimate the probability that a patient has the outcome of interest. After simplifying (as *A, G* and *BMI* proved to be of no influence), we first calculate for a patient with a particular *NRS*,

$$S = a + b_1 NRS \qquad (2)$$

Then, the probability that a patient has the outcome of interest is estimated as

$$p = \frac{e^S}{1+e^S} \qquad (3)$$

and the probability that a patient does not have the outcome of interest as

$$1 - p = \frac{1}{1+e^S} \qquad (4)$$

The probability *p* decreases from one to zero, for *S* decreasing from plus to minus infinity. Noticeably, equation (3) shows that the probability p=0.5 for S=0 or $NRS = -a/b_1$. Table S3.1 lists the estimated constant terms and logistic regression coefficients.

Table S3.1. Results from the logistic regression model using the 11-points Numerical Rating Scale for movement-evoked pain as explanatory variable for each of the three dependent variables PO, NO, and PONO. Listed are constant term *a* and logistic regression coefficient b_1 from equation (2), as well as the odds ratio (OR) with its 95% Wald confidence interval.

Day after surgery	Dependent variable	*a*	(SE)	b_1	(SE)	OR	(95% CI)
1	PO	4.1156	(0.0943)	-0.6039	(0.0166)	0.547	(0.529-0.565)
	NO	4.7171	(0.1120)	-0.7773	(0.0197)	0.460	(0.442-0.478)
	PONO	3.5161	(0.0871)	-0.6805	(0.0169)	0.506	(0.490-0.523)
2	PO	3.8677	(0.1243)	-0.5303	(0.0224)	0.588	(0.563-0.615)
	NO	5.0953	(0.1651)	-0.7912	(0.0288)	0.453	(0.428-0.480)
	PONO	3.4504	(0.1156)	-0.6220	(0.0224)	0.537	(0.514-0.561)
3	PO	3.4035	(0.1430)	-0.4578	(0.0275)	0.633	(0.600-0.668)
	NO	5.3441	(0.2248)	-0.8378	(0.0402)	0.433	(0.400-0.468)
	PONO	3.1292	(0.1364)	-0.5719	(0.0280)	0.564	(0.534-0.596)

PO, patient's opinion on whether the pain is acceptable; NO, nurses' observation on the patient's ability to make appropriate movements; PONO, combined measure of PO and NO: is "acceptable pain" associated with "good appropriate movements" or not.

The values in Table S3.1 can be used to calculate *S* (eq. 2) and the probabilities given in equations (3) and (4). For example, a patient has NRS=4 on day 1. The probability that this patient moves appropriately is 0.833, whereas the probability of not moving appropriately is 0.167. The odds of moving appropriately is $p/(1-p)$ = 0.833/0.167 = 4.99. Alternatively, combining equations (3) and (4) yields $p/(1-p) = e^S = e^{4.7171-0.7773x4} = 4.99$.

Although mathematically correct, we should not apply estimates on the probability scale to individual subjects like we did in the example. Each individual subject reporting NRS=4 either does or does not move appropriately. The estimated probabilities from a logistic regression model are best viewed as estimates of proportions in the underlying population. As a result, we better express the result for the example as: the estimated proportion of patients that move appropriately at NRS=4 is 0.833. A confidence interval for an estimated proportion can be calculated. We therefore refer to Hosmer and Lemeshow.[†]

The odds ratio (OR) in the example is calculated as follows. For an *NRS*=4, it is the estimated odds of moving appropriately for *NRS*=5 relative to the estimated odds of moving appropriately for *NRS*=4. As the odds with *NRS*=5 is $e^{4.7171-0.7773x5}$ = 2.30, the OR = 2.30/4.99 = 0.46. Alternatively, it can be shown that the OR = e^{b_1} = $e^{-0.7773}$ = 0.46, as *NRS* increases by one unit. If the OR is equal to one, then the two odds are the same. An OR > 1 indicates an increased odds of moving appropriately, and an OR < 1 indicates a decreased odds of moving appropriately, as *NRS* increases by one unit.

A measure of the model's ability to discriminate between those subjects who experience the outcome of interest *versus* those who do not is provided by the area under the Receiver Operating Characteristic (ROC) curve (Figure S3.1).[††] It plots sensitivity (true positive fraction of subjects) *versus* 1-specificity (false positive fraction of subjects) at all possible cutoff points on the NRS.[§] The 'optimal' cutoff point is found where the vertical distance between the curve and the line of identity is maximal (Youden's J-statistic).

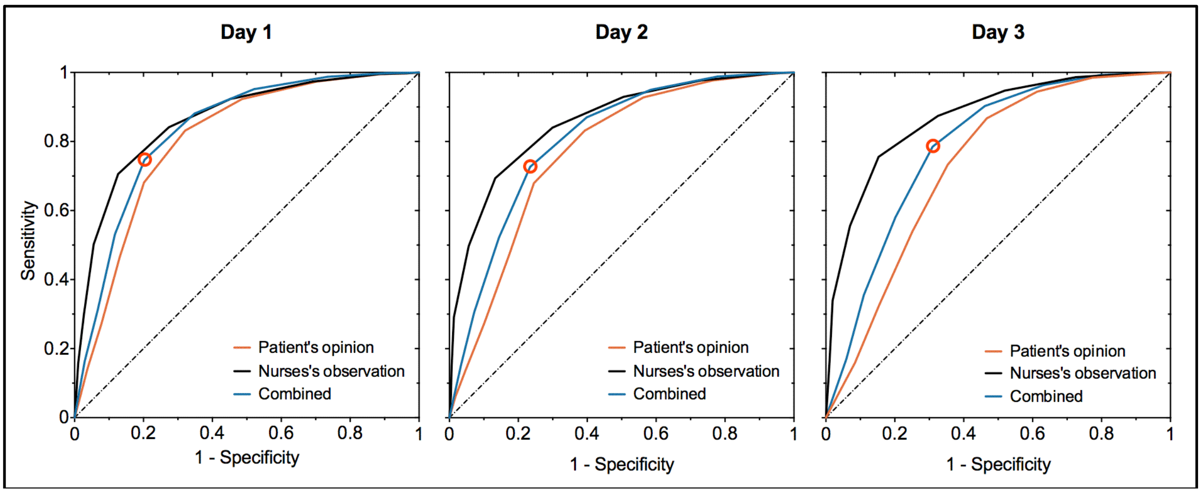

Fig. S3.1. ROC curves for the dependent variables PO, NO and PONO for the three first postoperative days. The dashed line is the line of identity where the AUC = 0.5. The closer the ROC curve is to the upper left corner, the better NRS discriminates. Open circles are the points where Youden's J-statistic is maximal for PONO.

[†]Hosmer DW and Lemeshow S. In: *Applied Logistic Regression*, 2nd Ed, Chapter 1, p.17-21. ISBN 0-471-35632-8.
[††]Hosmer DW and Lemeshow S. In: *Applied Logistic Regression*, 2nd Ed, Chapter 5, p.160-164.
[§] Galley H. Solid as a ROC. Editorial II. *Br J Anaesth* 2004; 93: 623-6. doi: 10.1093/bja/aeh247
